# Cortical Asymmetries during Hand Laterality Task Vary with Hand Laterality: A fMRI Study in 295 Participants

**DOI:** 10.3389/fnhum.2016.00628

**Published:** 2016-12-06

**Authors:** Emmanuel Mellet, Bernard Mazoyer, Gaelle Leroux, Marc Joliot, Nathalie Tzourio-Mazoyer

**Affiliations:** ^1^Groupe d’Imagerie Neurofonctionnelle, Institut des Maladies Neurodégénératives UMR 5293, Université BordeauxBordeaux, France; ^2^Centre National de la Recherche Scientifique, Groupe d’Imagerie Neurofonctionnelle, Institut des Maladies Neurodégénératives UMR 5293Bordeaux, France; ^3^Commissariat à l’Energie Atomique et aux Energies Alternatives, Groupe d’Imagerie Neurofonctionnelle, Institut des Maladies Neurodégénératives UMR 5293Bordeaux, France

**Keywords:** hand laterality task, hemispheric asymmetry, motor cortex, handedness, fMRI

## Abstract

The aim of this study was to characterize, using fMRI, the functional asymmetries of hand laterality task (HLT) in a sample of 295 participants balanced for handedness. During HLT, participants have to decide whether the displayed picture of a hand represent a right or a left hand. Pictures of hands’ back view were presented for 150 ms in the right or left hemifield. At the whole hemisphere level, we evidenced that the laterality of the hand and of the hemifield in which the picture was displayed combined their effects on the hemispheric asymmetry in an additive way. We then identified a set of 17 functional homotopic regions of interest (hROIs) including premotor, motor, somatosensory and parietal regions, whose activity and asymmetry varied with the laterality of the presented hands. When the laterality of a right hand had to be evaluated, these areas showed stronger leftward asymmetry, the hROI located in the primary motor area showing a significant larger effect than all other hROIs. In addition a subset of six parietal regions involved in visuo-motor integration together with two postcentral areas showed a variation in asymmetry with hemifield of presentation. Finally, while handedness had no effect at the hemispheric level, two regions located in the parietal operculum and intraparietal sulcus exhibited larger leftward asymmetry with right handedness independently of the hand of presentation. The present results extend those of previous works in showing a shift of asymmetries during HLT according to the hand presented in sensorimotor areas including primary motor cortex. This shift was not affected by manual preference. They also demonstrate that the coordination of visual information and handedness identification of hands relied on the coexistence of contralateral motor and visual representations in the superior parietal lobe and the postcentral gyrus.

## Introduction

The hand laterality task (HLT) consists in identifying if the hand presented in a picture is a left or a right one. It has been shown that in order to perform this task, participants implicitly mobilize a representation of their own right or left hand (Parsons, 1987b). Correspondingly, neuroimaging studies of HLT systematically reported activations in a fronto-parietal network including, premotor and superior parietal regions (Parsons et al., 1995; Kosslyn et al., 1998; Vingerhoets et al., 2002; Wraga et al., 2003; Seurinck et al., 2004; de Lange et al., 2005, 2006; Ferri et al., 2012; Zapparoli et al., 2014). Among these studies, only Kosslyn et al. (1998) reported an activation in primary motor cortex, while Berneiser et al. (2016) described that activity increased in the right primary motor cortex after an intensive training to HLT. This exemplifies the still on-going debate regarding its involvement HLT (de Lange et al., 2008; Hétu et al., 2013). Moreover, given that motor activity is one of the most strongly lateralized function in the brain, the left hand being controlled by the right motor cortex and vice-versa, it could be hypothesized that the asymmetry of activation in HLT varies according to the left or right handedness of the presented hand. Very few studies have so far reported a variation of functional asymmetries in motor, premotor or parietal regions according to the handedness of the presented hand during HLT. Only de Lange et al. (2006) using fMRI in a sample of 17 right-handers (RH), mentioned that activation of right premotor and right intraparietal sulcus was greater when a left hand was presented while their left counterpart was equally activated whichever the presented hand. In the same vein, a pioneering PET study included seven right-handed participants performing two conditions: left hands presented in the right visual field and right hands presented in the left visual field (Parsons et al., 1995). Asymmetries were not statistically tested but the authors reported that activation appeared purely contralateral to the handedness of the stimulus in the prefrontal and insular cortex, but no asymmetry was reported in hand sensorimotor area. Thus, to our knowledge, no work have reported that the lateralization of activation in hand motor areas during HLT was affected by the handedness of the stimuli. Such an observation would be a convincing hint that one actually mentally moves the same hand than the one to be identified during this task, supporting the embodied nature of HLT.

HLT can be performed using two strategies. One involves motoric processes by mobilizing a motor representation of one’s own right or left hand (Parsons, 1987b), the other is based on visuo-spatial mental rotation by rotating the picture of the presented hand. At the behavioral level, the motoric nature of the involved processes is supported by the fact that reaction times (RT) for identifying the laterality of the presented hand are longer when the hand is in an awkward position (typically away from the body midline) than when it is presented in a more familiar position, thus reproducing the biomechanical constraints of actual movements (Parsons, 1987a, 1994). Based on this behavioral signature, it has been shown that the perspective of the presented hands influences the strategy: palm views favor a motor imagery strategy while back views promote a visuo-spatial strategy (ter Horst et al., 2010; Bläsing et al., 2013). A recent meta-analysis has shown that strategies can be distinguished at the brain level (Tomasino and Gremese, 2015): the direct comparison between the motor and visual strategy evidenced activations in the postcentral gyrus while the reverse contrast revealed more important activations in occipito-temporo-parietal cortex. It is worth noticing that it has also been underlined that the dissociation between both strategies was not complete at the neural level: back views and palm view of hands give rise to largely overlapping activations while their processing is supposed to rely on visuo-spatial and motor strategy respectively (Zapparoli et al., 2014).

In their study, Parsons et al. (1995) reported that the posterior parietal cortex showed “a mix of visual, somatic and dominance lateralizations”. As a matter of fact, activation in the parietal cortex, and more specifically in the intraparietal sulcus and the superior parietal lobule, are consistently observed in HLT. They correspond in part to the visuo-spatial component of HLT. As the motor cortex, these regions are highly lateralized since left and right parietal cortices host a representation of the contralateral hemifield (Sereno et al., 2001; Swisher et al., 2007; Silver and Kastner, 2009). Moreover, these regions are key hubs for integrating motor and visual information and thus may be also sensitive to the laterality of the presented hand in addition to that of the hemifield. It is unknown however whether these two features are independent or interacting. We address this question in the present study using the same hemifield presentation design that Parsons et al. (1995) the left or right hand being briefly displayed in the left or right visual hemifield. One aim of our study was thus to assess, in a large sample of participants, the functional asymmetry of brain regions according to both handedness of stimulus and hemifield of presentation and to characterize the relationship between visual and motor laterality in regions that were sensitive to both.

It has been hypothesized that left-handers (LH) and RH might exhibit behavioral differences at HLT because of their different dominant hand. This hypothesis has been tested behaviorally in several works. Most of them reported that RH recognized faster a right hand than a left hand while no difference existed in LH (Gentilucci et al., 1998b; Ionta and Blanke, 2009; Ní Choisdealbha et al., 2011). At the brain level, one study dealing with explicit motor imagery has shown that LH and RH exhibited different patterns of activation in the precentral, central and post-central regions (Willems et al., 2009), but to our knowledge, no neuroimaging study has compared LH and RH during HLT. A second aim of the present study was thus to evaluate the effect of handedness on brain functional asymmetries during this task.

In summary, although several studies have documented the neural bases of HLT, a description of the asymmetries and the inter-hemispheric organization for this task and its variability according to handedness is still lacking, leaving open the issue of whether or not HLT neural support fits with the strongly lateralized organization of the hand motor system. The present work investigated, the variation of asymmetry in pairs of functional homotopic regions selected for their sensitivity to hand laterality in the large sample of LH and RH of the BIL&GIN database (Mazoyer et al., 2016). Based on the approach implemented by Parsons et al. (1995) the main goals of the study were: (1) to identify the regions sensitive to the handedness of the stimulus and to examine whether they include sensorimotor regions; (2) to characterize the variations of functional asymmetries according to hand laterality and visual hemifield in these regions; (3) to evaluate the effect of participants’ handedness on hemispheric and regional functional asymmetry of pairs of homotopic regions in a large sample of subjects balanced for handedness.

## Materials and Methods

### Participants

The study was approved by the Basse-Normandie local Ethics Committee CPP Nord-ouest III. All participants had no neurological history. Two-hundred and ninety-five healthy adults (mean age ± SD: 25.4 ± 6.0 years, 147 women,) provided informed written consent to participate in the study. Participants’ sample was balanced for handedness with 145 RH, 150 LH. The scores to the Edinburgh questionnaire ranged from −100 to 55 for LH and from 41 to 100 in RH.

### Data Acquisition

#### Experimental Condition

The paradigm consisted of 32 event-related trials lasting 12 s each, during which the subject had to decide the handedness of left and right hands in various orientation presented in either their left or right visual field. In order to induce variations in the strength of hemispheric recruitment and thus in hemispheric asymmetries, the experimental paradigm included four conditions: (1) left hands presented in the left visual hemifield (Lhand_Lhemi); (2) left hands presented in the right hemifield (LH and_Rhemi); (3) right hands presented in the left hemifield (Rhand_Lhemi); and (4) right hands presented in the right hemifield (Rhand_Rhemi). Each stimulus was presented for 150 ms with its closest edge at 1.5° of visual angle to the left or right of a central fixation crosshair. Each hand image subtended ~5° of visual angle. The left or right hand was oriented either clockwise 60° or 90°, or counterclockwise −120° or −90°. All eight stimuli (4 orientations × 2 hands) were presented in each visual hemifield in random order, resulting in 16 different stimuli repeated twice. In the MRI scanner, the participant was lying with his two hands resting on his thighs. Participants responded with one hand only but a fiber optic response pad (Current Designs Inc., Philadelphia, PA, USA) was placed in each hand in order to have their right and the left hand in the same position. Before the experiment start, participants were asked with which hand they preferred to respond. All 145 RH and 133 LH chose their right hand, while 17 LH chose their left hand. According to their preference, they had to respond with their right hand (resp. left hand) by pressing the right button of the response pad with their right middle finger (resp. left forefinger) to indicate a right-hand stimulus or the left button with their right forefinger (resp. left middle finger) for a left hand within the 2850 ms following the brief display of the hand. After the response, the central fixation crosshair was replaced by a square. After ~7 s the square was replaced by the fixation crosshair indicating that a new picture of hand will be soon displayed. The subjects were asked to continuously fixate the cross or the square presented at the center of the screen. Both fixation crosshair and square covered 0.8° × 0.8° of visual angle.

The experiment presentation was programmed in E-prime software (Psychology Software Tools, Pittsburg, PA, USA) integrated in the IFIS-SA system (MRI Devices Inc, Gainesville, FL, USA). Stimuli were then presented using an in-house adaptation of the IFIS-SA system and were projected from an Optoma EX330 DLP projector (Optoma Europe Ltd, Watford, UK) onto a translucent screen. Participants viewed a backlit projection coming from the rear of the magnet bore through a mirror mounted on the head coil. In order to limit the effects of a black background light emitted by the projector, the translucent screen was placed at the entrance of the scanner bore, closer to the subject so that he could not see any screen edge in the mirror.

The HLT task was part of the acquisition of a large database including seven other runs implementing tasks in various cognitive domains and was thus limited in the number of stimuli. A behavioral post-session HLT was performed on a laptop after the scanning to supplement behavioral data of the fMRI session. It followed the same design that in the scanner but included 48 stimuli from −150° to 150° by step of 30° (12 angles × 2 hands × 2 hemifield). The data of 269 subjects were available (131 LH). For left hand medial orientations correspond to 30°, 60°, 90°, 120° and 150° and lateral ones to −30°, −60°, −90°, −120° and −150° and the reverse for right hands.

Finally an additional behavioral experiment was achieved to assess the effect of unimanual or bimanual modality of response. The design was identical to the one of post-scanning session described above and 55 participants were included (27 LH). Unlike the above experiment, the response was bimanual, i.e., participants pressed a key with their left index when a left hand was presented and with the right index when a right hand was displayed.

#### Image Acquisition

Imaging was performed on a Philips Achieva 3 Tesla MRI scanner. Structural MRI protocols consisted of a localizer scan, a high resolution 3D T1-weighted volume (sequence parameters: TR = 20 ms; TE = 4.6 ms; flip angle = 10°; inversion time = 800 ms; turbo field echo factor = 65; sense factor = 2; field of view = 256 mm^3^ × 256 mm^3^ × 180 mm^3^; 1 mm^3^ isotropic voxel size) and T_2_*-weighted multi-slice images were also acquired (T2*-weighted fast field echo (T2*-FFE), sequence parameters: TR = 3500 ms; TE = 35 ms; flip angle = 90°; sense factor = 2; 70 axial slices; 2 mm^3^ isotropic voxel size). Functional images were acquired with a whole-brain T_2_*-weighted echo planar image acquisition (T_2_*-EPI, sequence parameters: 192 volumes; TR = 2 s; TE = 35 ms; flip angle = 80°; 31 axial slices; 3.75 mm^3^ isotropic voxel size) covering the same field of view as the T2*-FFE acquisition.

#### Image Processing

Image analysis was performed using the SPM5 software. The T1-weighted scans of the participants were normalized to a site-specific template (T-80TVS) matching the MNI space, using the SPM5 “segment” procedure with otherwise default parameters. So as to correct for subject motion during the fMRI runs, within each run, the 192 EPI-BOLD scans were realigned using a rigid-body registration. The EPI-BOLD scans then were registered rigidly to the structural T2-weighted image, which was itself registered to the T1-weighted scan. The combination of all registration matrices allowed warping the EPI-BOLD functional scans to the standard space with a trilinear interpolation. Once in the standard space, a 6-mm FWHM Gaussian filter was applied.

##### Hemispheric functional asymmetries

The BOLD signal was measured in the 192 pairs of homotopic Regions of Interest (hROIs) defined from intrinsic (resting-state) connectivity in the AICHA Atlas (Joliot et al., 2015), excluding 13 pairs of hROIS belonging to the orbital and inferior-temporal parts of the brain because of susceptibility artifacts. For each participant we computed for each of the four conditions and each of 179 remaining pairs an index of regional asymmetry as the difference between the left and right hROI BOLD signal variation for this pair during that condition. Finally, for each individual, the asymmetry indices of the 179 hOI pairs were averaged resulting in an individual hemispheric index of asymmetry.

##### Regional functional asymmetries

In addition, we selected among the 179 ROI pairs those exhibiting a significant difference in the contrast (Lhand_Lhemi + Lhand_Rhemi) *minus* (Rhand_Lhemi + Rhand_Rhemi) at *p* ≤ 0.05, Bonferroni corrected (corresponding to *p* ≤ 0.0001 uncorrected).

#### Statistical Analysis

Statistical analysis was conducted with the JMP software (SAS, Cary, USA, version 11.0) for behavioral data analysis and with R^1^ for the brain data analysis.

##### Behavioral data

As there was no difference between the 278 subjects who responded with their right hand and the 17 subjects who responded with their left hand, we pooled these two samples in the analysis of behavioral data.

In order to compare our behavioral results to those of Parsons (1987a, 1994), we first defined hand orientation as medial for angles of rotation toward the mid-sagittal plane (60° and 90° clockwise for left hand and 90° and 120° anti-clockwise for right hand) and as lateral for angles of rotation away the mid-sagittal plane (60° and 90° clockwise for right hand and 90 and 120° anti-clockwise for left hand).

We conducted a full factorial 2 × 2 × 2 × 2 ANOVA with hand laterality (Hand), presentation hemifield (Hemifield), and medial or lateral orientation (Orientation) as within-subject factors and Handedness as a between-subject factor.

##### Effects of the lateralization of the stimulus and of laterality factors on hemispheric functional asymmetry

The participants were analyzed separately according to their responding hand.

A first ANOVA included the 278 participants who responded with the right Hand. The hemispheric index of asymmetry was entered in an 2 × 2 × 2 ANOVA with Hand and Hemifield as within-subject factors and Handedness as a between-subject factor.

A second 2 × 2 ANOVA was conducted with the 17 participants who responded with the left hand including Hand and Hemifield as within-subjects factors.

##### Effects of the lateralization of the stimulus and of laterality factors on functional asymmetry between homotopic regions

The asymmetry between hROIs in the 278 right hand responders and the 17 left-hand responders were analyzed separately. In the former sample, Hand Hemifield and hROI were entered in an ANOVA with repeated measures as within-subject factors and Handedness as between-subject factor (2 × 2 × 17 × 2). In the latter sample only within-subjects factors were included given that all participants were left-handed (2 × 2 × 17 ANOVA).

## Results

### Behavioral Results (Figure 1)

#### fMRI Behavioral Data

The left- and right responders were pooled since the hand of response did not affect RTs (*F*_(1,295.8)_ = 0.15, *p* = 0.69).

**Figure 1:**
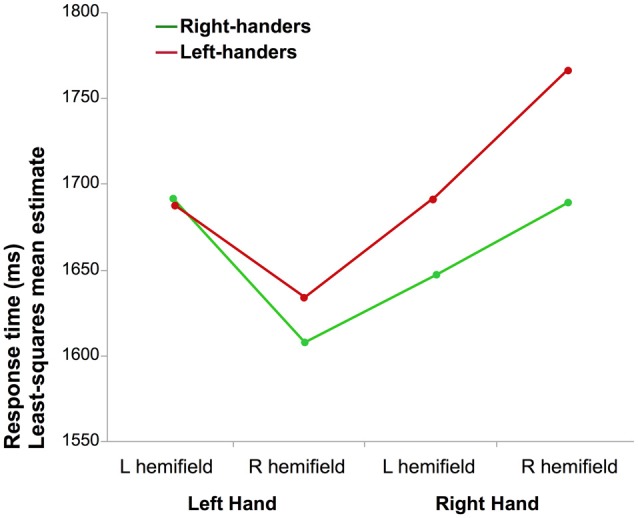
**Mean reaction time (RT) of correct laterality judgments.** The graph illustrates the interaction between the laterality of the hand and the hemifield in which it was presented for right-handers (RH) and left-handers (LH; the difference between both groups was not significant).

##### Reaction times (RT)

The results of the full factorial ANOVA revealed a main effect of Hand (*F*_(1,290.9)_ = 11.4, *p* = 0.0008, *η*^2^ = 4%) due to lower mean RT when a left hand was presented and a Hand × Hemifield interaction (*F*_(1,285.9)_ = 47.3, *p* < 0.0001, *η*^2^ = 14%). *Post hoc* analysis showed that participants responded faster for left hands than for right hands when presented in the right visual hemifield (*p* < 0.05, Tukey’s HSD). The results also showed a main effect of Orientation (*F*_(1,283.2)_ = 5.2, *p* = 0.02, *η*^2^ = 2%) medial orientation being identified faster than lateral, as well as an interaction between the Hand and Orientation factors (*F*_(1,274.8.1)_ = 99.1, *p* < 0.0001, *η*^2^ = 27%): corrected *post hoc* showed that medial orientation led to shorter RT than lateral for left hands while the opposite was observed for right hands (*p* < 0.05 for both). There was no effect of handedness (*F*_(1,290.9)_ = 0.33, *p* = 0.57). The Hand × Handedness interaction did not reach significance (*p* = 0.11).

##### Accuracy of responses

Response accuracy was significantly better for left hands (91 ± 11%) than for right hands (86 ± 13% *p* < 0.0001, Wilcoxon).

#### Post-Scanning Behavioral Data

##### Reaction times

A 2 × 2 × 2 ANOVA with repeated measures with Hand, Orientations as intra-subjects variables and Handedness as between-subjects factors was performed on the RT collected during the post-scanning session. It evidenced a main effect of orientation (*F*_(1,249.6)_ = 31.5, *p* < 0.0001, *η*^2^ = 11%) lateral orientations being processed faster than medial ones (Figure S1). There was a Hand × Orientation interaction (*F*_(1,248)_ = 9.1, *p* = 0.0029, *η*^2^ = 4%) showing that right hands were processed faster in lateral orientation than in medial one (*p* < 0.05, Tuckey’s HSD). No effect of participants’ handedness was detected.

##### Accuracy of responses

Response accuracy was significantly better for left hands (86 ± 13%) than for right hands (83 ± 15% *p* = 0.003, Wilcoxon).

#### HLT with Bimanual Responses

##### Reaction times

The same 2 × 2 × 2 as above evidenced a main effect of orientation (*F*_(1,50.68)_ = 7.0, *p* = 0.01 (*η*^2^ = 12%), lateral orientations being again processed faster than medial ones (Figure S2). There was however no Hand × Orientation interaction (*F*_(1,51.7)_ = 0.46, *p* = 0.5. There was a trend for a main effect of Hand (*F*_(1,52.7)_ = 3.1, *p* = 0.08), participants tending to provide faster responses for right hands. A Hand × Handedness interaction (*F*_(1,5.7)_ = 11.36, *p* = 0.001, *η*^2^ = 18%) evidenced that right hands were identified faster in RH (*p* < 0.05 Tuckey’s HSD) while no such difference exists in LH. Thus, when both hands are use to respond a right advantage in RH was found as described in previous studies.

##### Accuracy of responses

Response accuracy was significantly better for right hands (93 ± 8%) than for left hands (90 ± 10% *p* = 0.008, Wilcoxon).

### fMRI Results

#### Effect of Hemifield, Hand and Handedness on Asymmetry at the Hemispheric Level (Figure 2)

##### Participants responding with their right hand

There was a main effect of Hand (*F*_(1,276)_ = 67.71, *p* < 0.0001): identifying a left hand was associated with a more rightward lateralized than when identifying a right hand. A significant main effect of Hemifield was also observed (*F*_(1,276)_ = 206.75, *p* < 0.0001) due to a lower asymmetry index in case of a left hemifield presentation.

**Figure 2:**
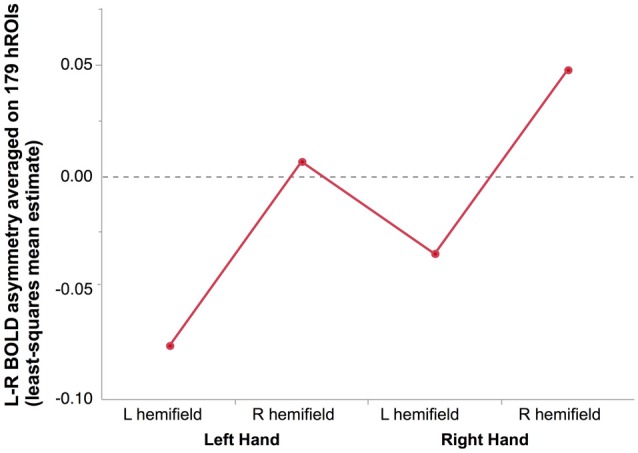
**Mean hemispheric asymmetry across the four conditions in 278 right-hand responders.** The asymmetry of 179 homotopic regions of the AICHA atlas was averaged in each subjects resulting in an individual hemispheric asymmetry index (Lhand_Lhemi: −0.08 ± 0.01; Lhand_RightHemi −0.01 ± 0.01; Rhand_Lhemi −0.04 ± 0.01; Rhand_Rhemi 0.05 ± 0.01).

As a result, the most negative asymmetry index value (reflecting a rightward asymmetry) corresponded to the condition supposed to appeal the most on the right hemisphere, namely presenting a left hand in the left hemifield, while the highest positive value corresponded the condition relying the most on the left hemisphere, namely presenting a right hand in the right hemifield (Figure 2).

There was no Hand × Hemifield interaction (*F*_(1,276)_ = 0.01, *p* = 0.91), no main effect of Handedness (*F*_(1,276)_ = 0.59, *p* = 0.44), and no Handedness × Hand nor Handedness × Hemifield interactions (*F*_(1,276)_ = 0.19, *p* = 0.66, *F*_(1,276)_ = 0.11, *p* = 0.74, respectively).

##### Participants responding with their left hand

The pattern was similar, to the one of the subjects who responded with the right hand. A main effect of hand was present (*F*_(1,16)_ = 9.27, *p* = 0.008 with a lower right asymmetry for right hands than for left hands. A main effect of Hemifield was also present (*F* *=*_(1,16)_ = 9.27, *p* = 0.006) with again no significant Hand × Hemifield interaction (*F*_(1,16)_ = 2.47, *p* = 0.14).

#### Identification of Regions Sensitive to Hand Laterality (Tables 1, 2, Figures 3, 4)

**Figure 3:**
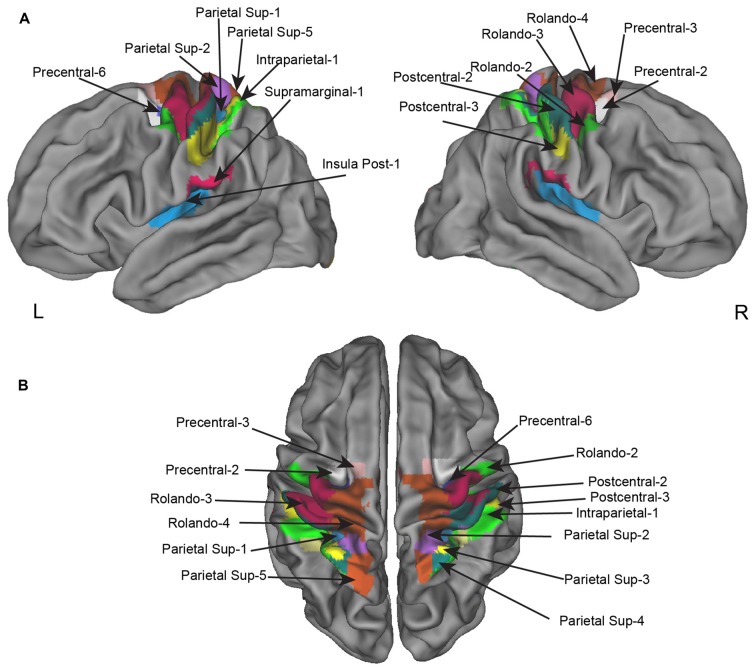
**Homotopic regions of the AICHA atlas included in the regional asymmetry analysis.** Seventeen regions have been selected as having significant BOLD variation in the left minus right hand contrast including four that had, in addition, a significant difference in the left minus right hemifield contrasts. **(A)** Left and right lateral view. **(B)** superior view.

**Figure 4:**
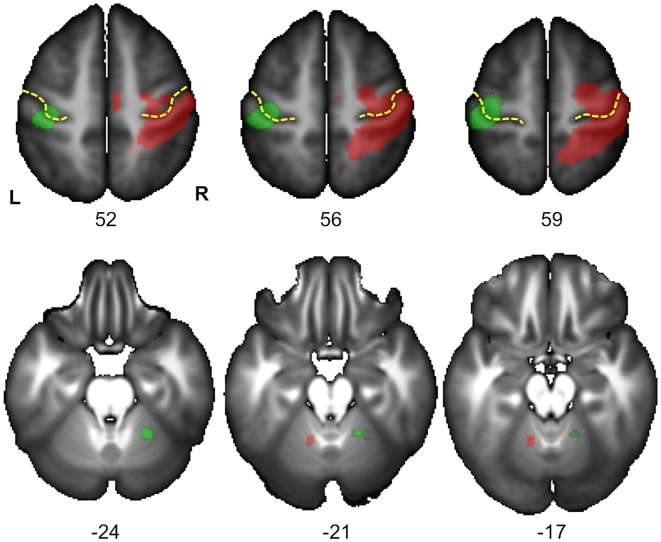
**Statistical parametric maps (SPM) for the right Hand *minus* left Hand (in green) and left Hand minus right Hand (in red) contrasts in the 278 subjects who responded with their right hand.** Maps are displayed at the threshold of *p* < 0.05 corrected (FWE) and superimposed on the BIL&GIN anatomical template (T1 weighted MRI averaged in 80 participants). In agreement with Figure 3 and Table 1, it displays a cluster of activation contralateral to the handedness of the presented hand in the sensorimotor cortex at the level of the hand representation including the primary motor cortex (Rolandic genu of the central sulcus in yellow). It additionally shows a cluster ipsilateral to the handedness of the presented hand in the cerebellum, in accordance with the cross-lateralized cerebral organization of motor activity (L: left, R: right, numbers correspond to the *z* coordinate of the axial slice).

We found 17 homotopic pairs of regions for which at least one element of the pair reached the significance threshold in the left Hand minus right Hand contrast (*p* < 0.0001, see Table 1). BOLD signal asymmetry for these 17 hROIs for the four conditions are listed in Table 2 and displayed in Figure 3. These regions belong to primary motor, premotor and somatosensory areas and also include the whole superior parietal lobule. Note that contrasting the left and right Hand conditions allowed to cancel out the activity related to the motor response since participants responded with the same hand in all conditions. The corresponding statistical parametric maps (SPM) maps are displayed in Figure 4.

**Table 1:** **Average BOLD signal variations of the left hand presentation minus right hand presentation contrast in the left and right hemisphere in the set of 17 homotopic regions of interest (hROIs) that showed a significant effect of hand**.

	Left hand minus right hand	
	Left hemisphere	Right hemisphere
S_Precentral-2	−0.09 (0.44)	0.23 (0.53)*
S_Precentral-3	−0.12 (0.49)*	0.22 (0.62)*
S_Precentral 6	−0.22 (0.59)*	0.29 (0.62)*
S_Rolando-2	−0.03 (0.62)	0.19 (0.58)*
S_Rolando-3	−0.30 (0.71)*	0.38 (0.61)*
S_Rolando-4	−0.10 (0.49)	0.21 (0.49)*
G_Paracentral_Lobule-1	−0.04 (0.60)	0.15 (0.55)*
S_Postcentral-2	−0.20 (0.65)*	0.38 (0.60)*
S_Postcentral-3	−0.08 (0.54)	0.19 (0.59)*
G_Insula posterior-1	−0.07 (0.53)	0.19 (0.66)*
G_Supramarginal- 1	−0.05 (0.60)	0.18 (0.67)*
G_Parietal_sup 1	−0.10 (0.49)	0.36 (0.56)*
G_Parietal sup-2	−0.10 (0.56)	0.36 (0.57)*
G_Parietal sup-3	−0.27 (0.71)*	0.42 (0.73)*
G_Parietal_sup-4	−0.09 (0.58)	0.22 (0.66)*
G_Parietal sup-5	−0.12 (0.64)	0.22 (0.64)*
S_Intraparietal-1	−0.05 (0.64)	0.21 (0.66)*

(Bonferroni correction for multiple comparisons *p < 0.0001, corresponding to p corrected for 358 tests).

**Table 2:** **Asymmetry (left minus right) in average BOLD signal variations in the 17 hROIs with a significant effect of Hand**.

	Lhand_Lhemi	Lhand_Rhemi	Rhand_Lhemi	Rhand_Rhemi
**Participants responding with the right hand**
S_Precentral-2	0.09 (0.05)	0.11 (0.05)	0.38 (0.05)	0.43 (0.05)
S_Precentral-3	0.09 (0.08)	0.10 (0.09)	0.42 (0.08)	0.44 (0.08)
S_Precentral 6	0.50 (0.09)	0.55 (0.09)	1.06 (0.08)	1.02 (0.09)
S_Rolando-2	1.40 (0.06)	1.43 (0.07)	1.63 (0.06)	1.61 (0.06)
S_Rolando-3	2.06 (0.08)	2.08 (0.08)	2.79 (0.07)	2.69 (0.07)
S_Rolando-4	0.55 (0.04)	0.60 (0.04)	0.87 (0.04)	0.89 (0.03)
G_Paracentral_Lobule-1	0.60 (0.04)	0.63 (0.05)	0.83 (0.04)	0.78 (0.04)
S_Postcentral-2	0.93 (0.06)	1.01 (0.06)	1.53 (0.06)	1.55 (0.06)
S_Postcentral-3	0.66 (0.05)	0.71 (0.05)	0.93 (0.05)	0.99 (0.05)
G_Insula posterior-1	0.57 (0.04)	0.54 (0.04)	0.82 (0.04)	0.82 (0.03)
G_Supramarginal-1	0.36 (0.05)	0.32 (0.05)	0.57 (0.05)	0.57 (0.05)
G_Parietal sup-1	−0.10 (0.06)	0.01 (0.06)	0.36 (0.06)	0.49 (0.06)
G_Parietal sup-2	−0.08 (0.06)	0.01 (0.06)	0.39 (0.05)	0.44 (0.05)
G_Parietal sup-3	0.59 (0.10)	0.85 (0.11)	1.32 (0.10)	1.50 (0.10)
G_Parietal sup-4	0.07 (0.07)	0.36 (0.07)	0.41 (0.06)	0.67 (0.07)
G_Parietal sup-5	0.12 (0.06)	0.28 (0.06)	0.44 (0.06)	0.64 (0.06)
S_Intraparietal-1	0.16 (0.06)	0.28 (0.06)	0.41 (0.06)	0.56 (0.06)
**Participants responding with the left hand**
S_Precentral-2	−0.75 (0.20)	−0.65 (0.19)	−0.35 (0.21)	−0.39 (0.17)
S_Precentral-3	−1.08 (0.40)	−1.24 (0.45)	−0.70 (0.36)	−0.73 (0.39)
S_Precentral 6	−0.95 (0.28)	−0.93 (0.36)	−0.16 (0.33)	−0.34 (0.28)
S_Rolando-2	−1.42 (0.17)	−1.39 (0.18)	−0.95 (0.17)	1.05 (0.20)
S_Rolando-3	−2.36 (0.17)	−2.26 (0.15)	−1.45 (0.18)	−1.44 (0.25)
S_Rolando-4	−0.81 (0.10)	−0.88 (0.12)	−0.51 (0.11)	−0.34 (0.12)
G_Paracentral_Lobule-1	−0.50 (1.00)	−0.48 (0.14)	−0.37 (0.13)	−0.31 (0.14)
S_Postcentral-2	−0.85 (0.22)	−0.91 (0.27)	−0.30 (0.20)	−0.19 (0.21)
S_Postcentral-3	−0.24 (0.26)	−0.35 (0.30)	−0.13 (0.21)	0.14 (0.21)
G_Insula posterior-1	−0.46 (0.10)	−0.49 (0.10)	−0.24 (0.11)	−0.29 (0.13)
G_Supramarginal-1	−0.80 (0.21)	−0.77 (0.21)	−0.45 (0.21)	−0.40 (0.22)
G_Parietal sup-1	−0.36 (0.22)	−0.55 (0.26)	−0.10 (0.21)	−0.05 (0.22)
G_Parietal sup-2	−0.58 (0.15)	−0.57 (0.14)	−0.23 (0.20)	0.05 (0.15)
G_Parietal sup-3	−0.21 (0.42)	−0.23 (0.45)	0.34 (0.40)	0.61 (0.44)
G_Parietal sup-4	0.15 (0.27)	0.26 (0.30)	0.24 (0.26)	0.45 (0.35)
G_Parietal sup-5	0.11 (0.25)	0.23 (0.27)	0.34 (0.30)	0.48 (0.25)
S_Intraparietal-1	−0.11 (0.31)	−0.17 (0.36)	−0.07 (0.30)	0.15 (0.32)

Mean (SE).

#### Effect of Hand Laterality, Hemifield of Presentation, and Handedness on the Functional Asymmetry of Homotopic Regions

##### Participants responding with the right hand

**Figure 5:**
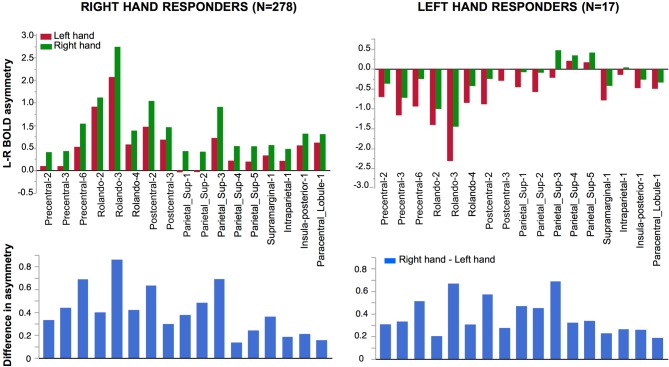
**Effect of Hand laterality on functional asymmetry in the 17 regions sensitive to hand laterality in right-hand and left-hand responders.** In the 17 pairs of homotopic regions of interest (hROIs) the leftward asymmetry was larger when a right hand was presented in right-hand responders (left superior corner). Symmetrically, in the group of left-hand responders, the rightward asymmetry was larger when a left hand was presented (right superior corner). Note that asymmetry Parietal 4–5 did not show a rightward asymmetry although participants used their left hand to respond. The lower part of the figure displays the asymmetry of the right hand *minus* left hand contrast (thus canceling the motor activity related to the response). The pattern of asymmetry appeared comparable in both groups.

Significant effect evidenced by the ANOVA with repeated measures on the 17 pairs of hROIs sensitive to hand laterality are reported in Table 3. In these regions, a main effect of Hand on their asymmetry index was present: the asymmetry was more leftward when participants identified a right hand than when they saw a left hand. (Figure 5, left column). Note that in these sensorimotor and premotor regions, there was an expected leftward asymmetry in all conditions related to the button press used for right hand response. *Post hoc* tests exploring the Hand × hROI interaction indicated that the hROI Rolando-3, corresponding to the hand primary motor area, exhibited the largest Hand effect (*p* < 0.05).

**Table 3:** **Results of the ANOVA with repeated measures on the asymmetry of the 17 hROIs selected as showing a difference between right and left hand laterality judgment on their right or left BOLD values**.

	*F*	*p*	df	partial *η*^2^
**Sample of right-hand responders**
Hand	435.1	*p* < 0.0001	276	61%
Hemifield	39.0	*p* < 0.0001	276	12%
hROI	110.8	*p* < 0.0001	4416	29%
Hand × hROI	42.4	*p* < 0.0001	4416	13%
Hemifield × hROI	18.4	*p* < 0.0001	4416	6%
Handedness × hROI	2.2	*p* = 0.004	4416	2%
**Sample of left-hand responders**
Hand	29.0	*p* < 0.0001	16	64%
hROI	5.9	*p* < 0.0001	256	27%
Hand × hROI	4.9	*p* < 0.0001	256	23%

*Participants were analyzed separately according to the hand they responded with. *F* value, *p* value, degree of freedom (df) and partial *η*^2^ of the significant main effects and interactions on the asymmetry the 17 homotopic regions are provided (df, degree of freedom)*.

**Figure 6:**
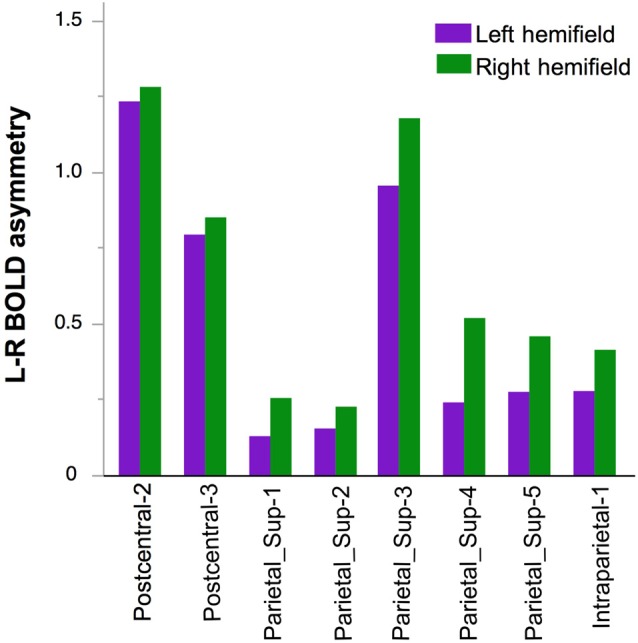
**Effect of Hemifield on functional asymmetry in the two postcentral and parietal hROIs in the 278 right-hand responders.** In these eight regions, the leftward asymmetry was stronger in case of right hemifield presentation.

A main effect of Hemifield on asymmetry was also observed (Figure 6). It differed across the regions as indicated by a significant Hemifield × hROI interaction. *Post hoc* analyses indeed showed that in eight regions, a left hemifield presentation was associated with reduced leftward asymmetry and vice-versa. These regions were the whole superior parietal lobule including Parietal-1, Parietal-2, Parietal-3, Parietal-4, Parietal-5 and Intraraparietal-1 in the most anterior part of the intraparietal sulcus (*p* = 0.002 for parietal-2 and *p* < 0.0001, for all the other regions, corrected for multiple comparison). In addition, asymmetry in two sensorimotor regions namely the Postcentral-2 (*p* = 0.04), the Postcentral-3 (*p* = 0.02), were also sensitive, in the same direction, to the hemifield of presentation.

The independence between Hand and Hemifield effects was attested by the absence of significant Hand × Hemifield × hROI and Hand × Hemifield interaction (*F*_(16,4416)_ = 1.34, *p* = 0.16 and *F*_(1,276)_ = 0.95, *p* = 0.33.

**Figure 7:**
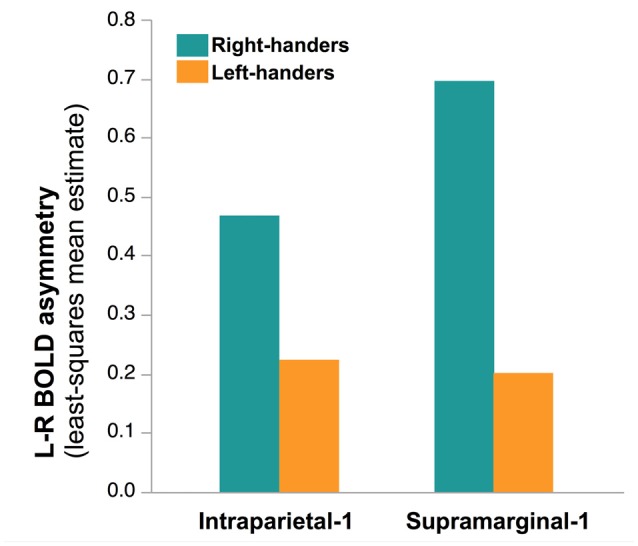
**Effect of Handedness on asymmetry of functional activity in the two hROIs that showed a main effect of handedness in the 278 right-hand responders.** In these two regions, located in the most infero-anterior part of the supramarginal gyrus and in the intraparietal sulcus, sensitive to hand laterality, the *post hoc* analysis of the Handedness × hROI interaction shows that LH were less leftward lateralized than RH. This effect was independent of the laterality of the hand stimulus.

Finally, no main effect of Handedness was observed (*F*_(1,276)_ = 0.81, *p* = 0.37) but a Handedness × hROI interaction was significant. *Post hoc* tests (corrected) showed that in two regions, namely Supramarginal-1 (*p* < 0.0001) and Intraparietal-1 (*p* = 0.04), RH were more leftward lateralized than LH (Figure 7). This was true whatever the hemifield and the laterality of the presented hand, since no Handedness × hROI × Hand nor Handedness × hROI × Hemifield was present.

##### Participants responding with the left hand

Significant effect evidenced by the ANOVA with repeated measures on the 17 pairs of hROIs sensitive to hand laterality in this subsample are reported in Table 3. A main effect of hand was present (Figure 5, right column). The asymmetries were more rightward when a left hand was presented. This effect differed according to the hROIs as indicated by the Hand × hROI interaction: a hand effect was significant in Rolando-3, Precentral-6, Postcentral-2 and Parietal_sup-3. In this much smaller sample, no Hemifield or Hemifield × hROIs was significant. No Hand × Hemifield was present either. The lower part of the Figure 4 shows that the direction and amplitude of asymmetry in the right hand minus left hand contrast was similar in both left and right hand responders.

## Discussion

### Methodological Considerations

As in some previous works (Vingerhoets et al., 2002; Seurinck et al., 2004; de Lange et al., 2005), we chose to keep the same hand of response whichever the handedness of the stimulus. This experimental choice was driven by the concern of preserving one hemisphere from any activity in the sensorimotor cortex related to the key press of the response pad. This made optimal the detection of a potential activation of primary motor cortex in the hemisphere ipsilateral to the response hand and allowed canceling out the activation related to the key press when comparing responses to presented left and right hands.

The HLT of the present work was one of the eight runs of the BIL&GIN database (Mazoyer et al., 2016). As a consequence, although the number of participants is very large as compared to other HLT studies, the number of hand stimuli processed by each participant is quite small. Because one of the main aims of the present study was to characterize the effect of the stimulus handedness on the functional asymmetries, rather than the effect of orientation and posture, the stimuli included four rotations and were restricted to back views of hands.

### Behavioral Results

Considering the main effect of orientation, we found that medial orientations were identified more quickly than lateral orientations in agreement with the previously “medial over lateral” effect (Parsons, 1987b, 1994). However, in participants who responded with their right hand, we found that left hand stimuli were identified faster in medial orientations while right hands was identified faster in lateral orientations. Our results thus did not show up a full “medial over lateral” advantage, that is shorter RT when hands are rotated toward the body’s midline as compared with rotations away from the body’s midline. This was also true for the post-scanning session and for the additional experiment including a bimanual response (Figures S1, S2). Considering that the motor imagery component appears all the more important than the number of axes of rotation is high (ter Horst et al., 2010), the fact that only back views of hands were included may have favor a visuo-spatial strategy. However, one should note that such a design did not prevent the involvement of sensorimotor areas as discussed below.

The present work reported shorter RT for left hands, as opposed to some previous studies that reported an advantage in right hand recognition (Gentilucci et al., 1998b; Ionta and Blanke, 2009; Takeda et al., 2010; Ní Choisdealbha et al., 2011). A previous work has however reported that RT for left hands were shorter when participants responded with the right hand (Cocksworth and Punt, 2013). Moreover, in the additional behavioral experiment we conducted, in which participants responded with left and right hand, we found a right-hand advantage. It thus likely that unimanual response altered the difference between left and right hands. This result has to be paralleled with the absence of difference between LH and RH observed in the behavioral data collected during the fMRI acquisition. On the opposite, the bimanual version of the experiment showed that RH responded faster for right hands than for left hands, while no difference was observed in LH. This last results is in agreement with a previous studies that also used a bimanual response (Gentilucci et al., 1998b; Takeda et al., 2010). However, responding with both hands confounds the advantage to respond with the dominant hand and the effect of familiarity of this dominant hand. As a matter of fact, whatever the task, RH respond faster with their dominant hand while response times are less affected by the side of the response hand in LH (Rabbitt, 1978; Tzourio-Mazoyer et al., 2015). Two previous studies overcame this problem by including a response with the ipsilateral foot (Ní Choisdealbha et al., 2011) or an oral response (Ionta and Blanke, 2009). These works also reported faster responses for right hands in RH while LH exhibited no difference. However, in these two studies both groups were analyzed separately and no group comparison was reported. The behavioral difference between LH and RH in HLT appears thus to be not fully clarified and seems to depend on response modality (Cocksworth and Punt, 2013).

### Effect of Hand Laterality and Presentation Hemifield on Hemispheric and Regional BOLD Variation Asymmetry

One goal of our HLT paradigm was to assess how handedness of a visually presented hand and the hemisphere in which it is presented modulate the functional asymmetry at both the hemispheric and regional level. Our results clearly show that these two types of lateralization combined their effects on modifying the hemispheric asymmetry at both levels. The absence of interaction between these two effects further suggests that they are purely additive. At the hemispheric level, the most rightward asymmetry corresponded to the additive effects of the lateralized processes allowing the identification a left hand flashed in the left hemifield, whereas the most leftward asymmetry was observed when a right hand was presented in the right hemifield. When the laterality of hand and that of the presentation hemifield were opposite, their effects on functional asymmetry subtracted from each other and led to in-between values of functional asymmetry. Note that the fact that participants responded with the right hand in all conditions did not mask the shift in hemispheric functional asymmetry related to the hand identification.

At the regional level, the present study evidenced a set of frontal and parietal regions the activity of which during HLT were sensitive to the hand stimulus laterality. These regions included the precentral, central and postcentral gyrus corresponding to the lateral premotor, primary motor and somatosensory cortex, respectively, all regions being involved in motor execution hand and for most of them in HLT (de Lange et al., 2006; Zapparoli et al., 2014; Berneiser et al., 2016). To our knowledge, this is the first evidence that activity in the primary motor cortex is affected by the laterality of the presented hand during HLT, what contributes to demonstrate the embodied nature of HLT at the brain level. Note that such pattern of asymmetry has been described during explicit motor imagery in primary motor and sensory cortex (Michelon et al., 2006). Moreover, in the present work, and similar to the study by Michelon et al. (2006) not only the hand primary motor area showed a difference in asymmetry with hand presentation in the present experiment, but the whole brain approach also evidenced a significant difference in the cerebellar area ipsilateral to the presented hand confirming the involvement of motor system during HLT (see Figure S1). Thus, although some behavioral studies concluded that identifying the laterality of hands from their back views rely on a visuo-spatial strategy rather than a motor strategy (ter Horst et al., 2010; Bläsing et al., 2013), and despite the absence of medial over lateral effect in three different sets of behavioral data collected here, the brain imaging results of the present study support that some motoric processes are actually involved in the laterality judgment of hands viewed from their back. It has been proposed that motor imagery is only confirmatory and was preceded by an automatic visual analysis allowing the identification of handedness (Parsons, 1994; Gentilucci et al., 1998b). Gentilucci proposed that this first step relied on an internal model derived from motor experience (Gentilucci et al., 1998a). This initial step might have thus contributed to the activation of motor and somatosensory areas observed in the present work even though the hand perspective did not subserve motor imagery. This is in agreement with the fact that, although processing palm views of hand might exhibit a greater dependence on motoric processes, it leads to activations that largely overlap the ones elicited by the processing of back views (Zapparoli et al., 2014).

The regions sensitive to hand stimulus laterality also encompassed the whole superior parietal lobule and a part of the intraparietal sulcus. These areas have been consistently involved in previous HLT neuroimaging studies (for a review, see Hétu et al., 2013). But our study also uncovered in these regions an asymmetry favoring the cortex contralateral to the laterality of the presented hand. This finding extends those of previous works in showing that shift of asymmetries during HLT related to the laterality of the hand presented. All these regions exhibited a strong effect of the hand laterality on their asymmetries, the hROIs Rolando-3 showing the largest effect. This region is located in the superior part of the Rolandic sulcus overlapping the *genu* of the central sulcus, an anatomical marker of the hand primary motor cortex (Rumeau et al., 1994). This result argues for a real involvement of the primary motor cortex in HLT, a claim that is still contentious since activation in this area during motor imagery has been inconstantly reported (Hétu et al., 2013).

Strikingly, the homotopic functional asymmetries of regions constituting the superior parietal lobule was, as the motor and sensorimotor cortex, markedly associated with the identification of the contralateral hand. Besides confirming the involvement of the superior parietal lobule in HLT, the present result is in line with the fact that patients bearing lesions restricted to the parietal cortex are selectively impaired at using a mental representation of the hand contralateral to the lesion side (Sirigu et al., 1996). In addition, the functional asymmetry of the superior parietal lobule, together with that of the anterior part of the intraparietal sulcus, was associated with the hemifield in which the hands were presented. The contralateral representation of the hand and of the hemifield acted on the asymmetry in an additive way and in the same direction. Interestingly, a similar visual/motor alignment effect has been described in a pointing task: pointing a target displayed in the right hemifield with the right hand elicits a larger activation in the left parietal lobe than pointing to this target with the left hand, with mirrored results in the right parietal (Medendorp et al., 2005). Our results thereby demonstrate that the coordination of visual information and motor imagery relies on the coexistence of contralateral motor and visual representations in the superior parietal lobe. In addition, we show that this inter-hemispheric organization extends outside the posterior parietal in two regions within the post-central gyrus including the primary somatosensory cortex, the asymmetry of which was associated with the presentation hemifield.

The asymmetry of the supramarginal gyrus-1 hROI was also associated with the hand laterality. In the AICHA atlas, this part of the supramarginal gyrus corresponds to the parietal operculum which has been identified as the human secondary somatosensory cortex (Eickhoff et al., 2006). This region, together with the postcentral gyrus, reflects the involvement of somatosensory cortex contralateral to the hand laterality during HLT what further supports that identification of hand laterality bears on the inter-hemispheric organization of actual hand motor activity. The posterior insular cortex, adjacent to supramarginal-1 has also been involved in somatosensory processing (Augustine, 1996), and more recently implicated in body scheme representation (Karnath and Baier, 2010). Our result suggests that as motor and somatosensory cortex, posterior insula codes for the contralateral hand.

### Effect of Handedness

One purpose of the present work was to investigate the effect of handedness on functional asymmetry during HLT. Regarding actual motor activity, it is has previously been shown that the motor cortex of LH and RH differ at both the anatomical and functional levels. In RH the left precentral sulcus is deeper as compared to its right counterpart, while the reverse pattern is observed in LH (Amunts et al., 1996). At the functional level, a more important lateralization contralateral to finger movements has been reported in RH compared to LH (Solodkin et al., 2001), while a more recent work underlined differences in deactivation of the ipsilateral cortex (Tzourio-Mazoyer et al., 2015). However, no effect of handedness was observed on functional asymmetries of motor and premotor areas in the present work. This finding echoes the lack of difference in activation related to handedness in these regions during actual finger movement (Tzourio-Mazoyer et al., 2015). In this previous work, we indeed showed using the same sample of participants that the difference between LH and RH during right and left finger tapping was not in activation strength but in the amplitude of the deactivation of the ipsilateral motor cortex during the dominant hand movement. Such absence of effect of handedness in HLT contrasts with a previous report by other investigators that LH and RH exhibited different patterns of activation during explicit motor imagery in precentral, central and post-central regions (Willems et al., 2009). In this latter study, participants had to imagine manual motor action cued from manual action verbs without instructions regarding the hand to be imagined. It is thus likely that each handedness group used its dominant hand to perform manual actions and thus activated the sensorimotor areas contralateral to their dominant hand. This could explain why regions showing a handedness effect in this latter study largely overlapped the regions that exhibited a hand laterality effect in the present report.

Handedness had no effect on functional asymmetry at the hemispheric level either and was actually restricted, at the regional level to a couple of hROI : a part of the supramarginal gyrus corresponding to the secondary somatosensory cortex and to the most anterior part of the intraparietal sulcus. In these areas, RH were more leftward lateralized than LH. This effect thus concerned only a narrow part of the cortex and did not depend on the characteristics of the stimulus such as the laterality of the hand and or the presentation hemifield since no interaction was observed between handedness and these two factors. This relatively limited effect at the brain level is consistent with the weak effect of handedness observed at the behavioral level. It remains to establish whether this effect is specific of HLT or whether it reflects a difference between LH and RH across various tasks.

## Clinical Implications

Besides its suitability for investigating embodiment of the mental representation of body parts, the HLT has gained a substantial clinical importance. It has for example been proposed as a tool for assessing pain intensity in the complex regional pain syndrome (Schwoebel et al., 2002).This last study reported slower RT for affected than unaffected limb. It also suggested from previous neuroimaging studies on HLT that pain induced alteration of body schema is mediated by posterior parietal rather the primary motor cortex. The present work supports that primary motor and sensory cortex were actually involved the body schema. These regions could thereby also play a role in the increase of RT of the painful limb.

Explicit motor imagery has also been proposed to be included in motor rehabilitation programs after a stroke (Sharma et al., 2006; Braun et al., 2008). The fact that HLT activates motor cortex in a lateralized way, even when back views of hand are used, could make it suitable for that purpose, either at the initial phase of rehabilitation or in complement to explicit motor imagery tasks. The instructions of HLT are simple, making this task less dependent on patients’ compliance or understanding than more complex motor imagery tasks. The laterality of the presented hand should also be considered, as it determines the side of the motor cortex that will be more involved in the task. This is true whichever the handedness of the patient as LH and RH did not exhibit any differences in the motor cortex.

## Conclusion

Thanks to the evaluation of asymmetry in homotopic regions, we showed here that HLT exhibit the same shift in asymmetry than actual hand movement in premotor, motor and somatosensory areas. We extended the results of Parsons et al.’s (1995) study, in showing that the processing of visual and motor information in HLT relies on the coexistence of contralateral motor and visual representations in the superior parietal lobe that led to independent shift of asymmetry unaffected by handedness. It remains to be determined whether the regional effect of handedness evidenced here is specific to HLT, an investigation that we are currently conducting using the BIL&GIN database.

## Author Contributions

EM conceived the research acquired the neuroimaging and behavioral data, performed data analyses and wrote the article; BM and NT-M acquired neuroimaging and behavioral data, performed data analyses and wrote the article; GL and MJ performed data analyses.

## Conflict of Interest Statement

The authors declare that the research was conducted in the absence of any commercial or financial relationships that could be construed as a potential conflict of interest.

## Footnotes

^1^https://www.r-project.org

## Supplementary Material

Figure S1 **Mean response time (RT) by stimulus orientation in the post-scanning behavioral session.** Anticlockwise angles of rotation are negative and corresponded to lateral orientations for left hand (in red) and medial orientations for right hand (in green). RT increase with the amplitude of the angles of rotation and are shorter for lateral than medial orientations. Bars error represent the 95% confidence interval.

Figure S2 **Mean response time by stimulus orientation in the hand laterality task (HLT) session with bimanual responses.** See Figure S2 for legend.
