# Supplementary figures and images for: Cortical Asymmetries during Hand Laterality Task Vary with Hand Laterality: A fMRI Study in 295 Participants
Source: Front Hum Neurosci. 2016 Dec 6;10:628. doi: 10.3389/fnhum.2016.00628 (PMC5138568; doi:10.3389/fnhum.2016.00628)

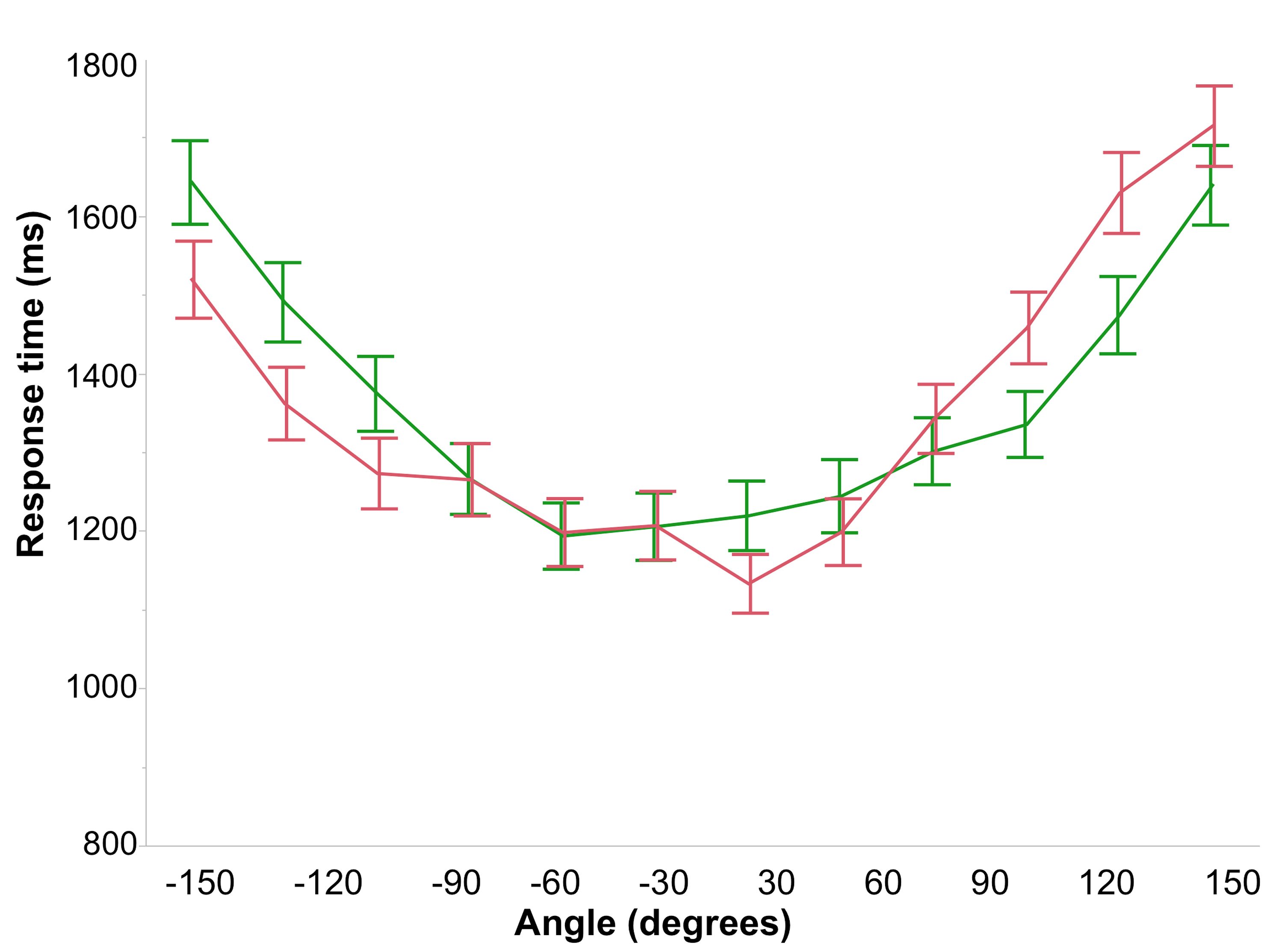

Supplement: Figure S1 — Mean response time (RT) by stimulus orientation in the post-scanning behavioral session. Anticlockwise angles of rotation are negative and corresponded to lateral orientations for left hand (in red) and medial orientations for right hand (in green). RT increase with the amplitude of the angles of rotation and are shorter for lateral than medial orientations. Bars error represent the 95% confidence interval. [file Image_1.jpeg]

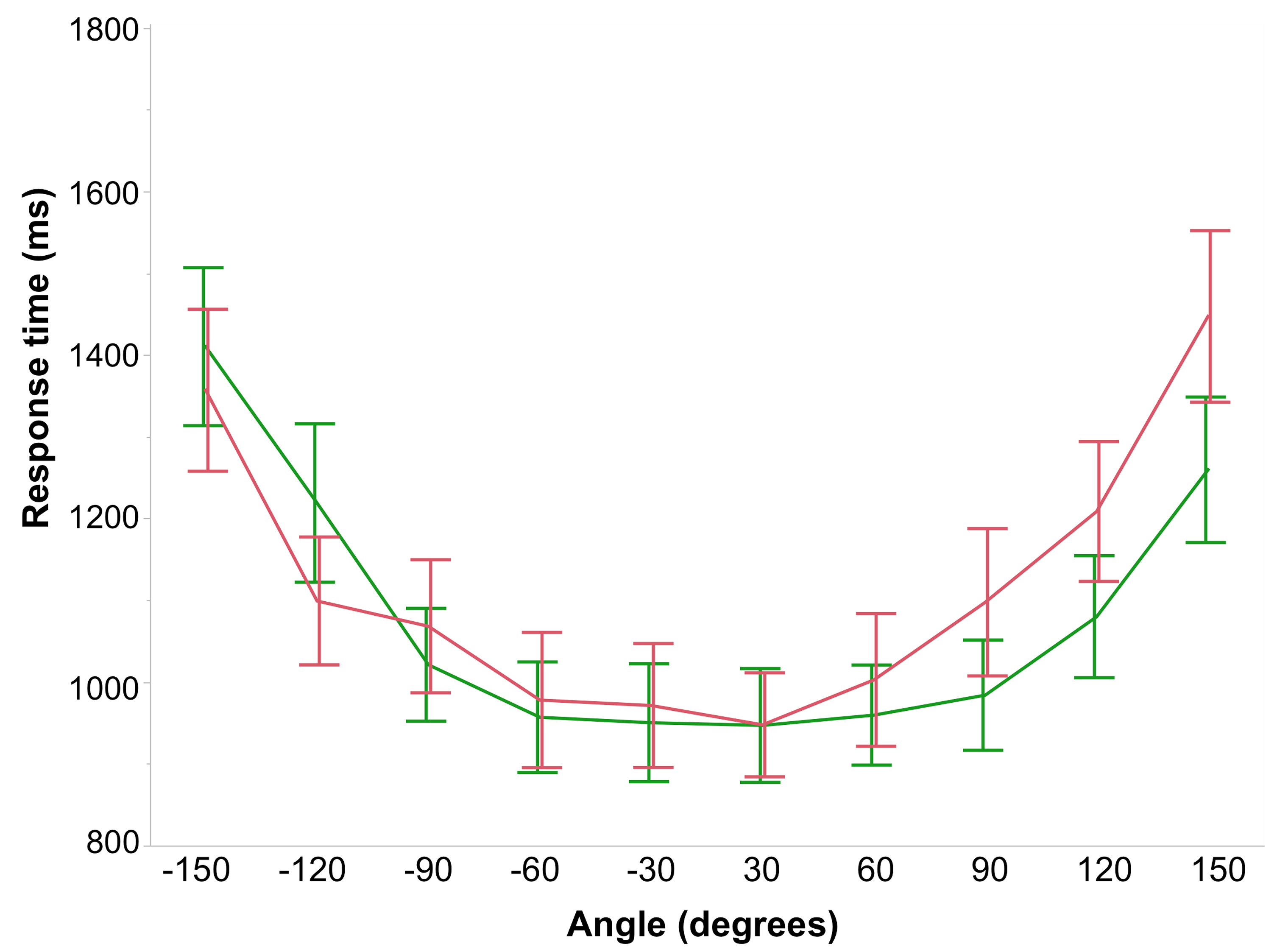

Supplement: Figure S2 — Mean response time by stimulus orientation in the hand laterality task (HLT) session with bimanual responses. See Figure S2 for legend. [file Image_2.jpeg]
